# Novel chemical route for CeO_2_/MWCNTs composite towards highly bendable solid-state supercapacitor device

**DOI:** 10.1038/s41598-019-42301-y

**Published:** 2019-04-10

**Authors:** Bidhan Pandit, Babasaheb R. Sankapal, Pankaj M. Koinkar

**Affiliations:** 1 Nano Materials and Device Laboratory, Department of Physics, Visvesvaraya National Institute of Technology, South Ambazari Road, Nagpur, 440010 Maharashtra India; 2 Department of Optical Science, Tokushima University, 2-1 Minamijosanjima Cho, Tokushima, 7708506 Japan

## Abstract

Electrode materials having high capacitance with outstanding stability are the critical issues for the development of flexible supercapacitors (SCs), which have recently received increasing attention. To meet these demands, coating of CeO_2_ nanoparticles have been performed onto MWCNTs by using facile chemical bath deposition (CBD) method. The formed CeO_2_/MWCNTs nanocomposite exhibits excellent electrochemical specific capacitance of 1215.7 F/g with 92.3% remarkable cyclic stability at 10000 cycles. Light-weight flexible symmetric solid-state supercapacitor (FSSC) device have been engineered by sandwiching PVA-LiClO_4_ gel between two CeO_2_/MWCNTs electrodes which exhibit an excellent supercapacitive performance owing to the integration of pseudocapacitive CeO_2_ nanoparticles onto electrochemical double layer capacitance (EDLC) behaved MWCNTs complex web-like structure. Remarkable specific capacitance of 486.5 F/g with much higher energy density of 85.7 Wh/kg shows the inherent potential of the fabricated device. Moreover, the low internal resistance adds exceptional stability along with unperturbed behavior even under high mechanical stress which can explore its applicability towards high-performance flexible supercapacitor for advanced portable electronic devices.

## Introduction

Significant research on supercapacitors (SCs) is targeted at accumulative power and energy density; however, recently attention is also focused at lowering manufacture costs and using environmental friendly materials too. To fulfill these requirements, the electrode materials should have high surface area and narrow pore size distribution while providing easy access of electrolyte ions to exhibit high energy storage capacity and good stability. To fabricate such electrode for electrochemical SCs, the electroactive materials should offer efficient mass transport, highly accessible electrochemical surface area and good electron conductivity which cannot be supplied solely by the individual electroactive material^1^.

To address the above mentioned activities, carbonaceous materials (such as activated carbon, carbon nanotube, graphite oxide, and reduced graphite oxide) have been drawn much attention with good electrode conductivity but limited to higher specific capacitance. In fact, hierarchically structured carbon-based composites with faradaic pseudo-capacitive system could be better candidates for SCs offering larger energy density and higher power density. Such composites combine an electric double layer capacitive (EDLC) and a faradaic pseudo-capacitive systems through nanoarchitecturing. This approach takes advantages as both utilize the fast and reversible faradaic capacitance coming from the electroactive pseudocapacitive species and the indefinitely reversible double-layer capacitance at the electrode-electrolyte interfaces. Carbon nanotube (CNT) has a unique set of properties including high mechanical properties, namely tensile strength and elastic modulus, and still remarkable flexibility, excellent thermal and electric conductivities (10^2^−10^5^ S/cm)^2^, low percolation threshold through loading weight at which a sharp drop in resistivity occurs and high aspect ratios through length to diameter ratio (L/D). Besides being responsible for high conductivity, the delocalized π-electrons of carbon nanotubes could be utilized to promote adsorption of various moieties on the CNT surface via π–π stacking interactions^3^. Although highly desirable, it is a great challenge to design and synthesize unique carbon based composites with hierarchical nanostructures in a controllable and much simpler manner, which can tailor the physical and chemical properties of the electroactive materials to meet the basic requirements for the supercapacitor applications.

**Figure 1:**
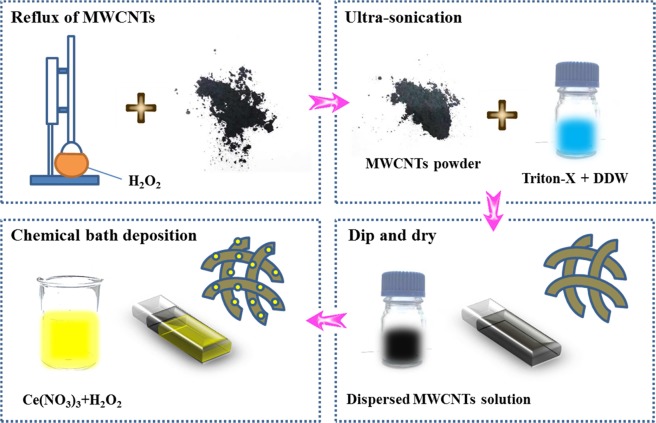
Schematic for the deposition of CeO_2_ nanoparticles on MWCNTs.

In this feature article, we have designed and fabricated flexible symmetric solid-state supercapacitor (FSSC) device using CeO_2_/MWCNTs electrodes sandwiched by PVA-LiClO_4_. Initially, CeO_2_ nanoparticles were deposited on MWCNTs surface to form composite nanostructure using simple, low-cost and environment-friendly chemical bath deposition (CBD) method (Fig. 1). There are a few literatures available regarding the supercapacitive performance of CeO_2_-MWCNTs composite electrodes. Kalubarme *et al*. prepared carbon nanotube (CNT)/cerium oxide composite and gained specific capacitance of 289 F/g^4^. Deng *et al*. reported a specific capacitance of 455.6 F/g at specific current of 1 A/g for CeO_2_/MWCNTs nanocomposite^5^. Luo *et al*. synthesized CeO_2_/CNTs hybrid electrode through hydrothermal method to achieve a maximum specific capacitance of 818 F/g at scan rate of 1 mV/s^6^. Nanostructured CeO_2_ can improve the transport and redox properties along with improved surface to volume ratio compared to other bulk and nanostructured materials. By considering nanocryatalline electroctive metal oxides, the energetics may be substantially reduced due to defects and increases the nonstoichiometry levels and electronic carrier generation^7^. The fluorite-structured cerium oxide forms a close packed structure array of atoms with four coordinate O^2−^ and eight coordinate Ce^4+^. As inexpensive and environmentally abundant rare earth element, CeO_2_ draws most attention with a ground state valance of 4f^1^5d^1^6s^2^ and hence, it not only forms steady Ce^3+^ by losing one 5d and two 6s electrons but stable Ce^4+^ by losing one extra 4f electron^8^. The oxidation state can be easily changed between Ce^3+^ and Ce^4+^, making CeO_2_ as an outstanding redox material as required for supercapacitor application. The efficient characteristics such as structural defects depending upon the partial pressure of oxygen and high mobility of oxygen vacancies can show the potential in energy-related application^9^. Hence, the assembly in combination with MWCNTs and nanostructured CeO_2_ opens new perspectives for the development of electrode material towards high-performance SCs including the following options: (i) exhibits sufficient electrical conductivity; (ii) increases the contact surface area between the electrode and the electrolyte; (iii) protects the electrolyte from decomposition by catalytic reactions with the electrodes; (iv) decreases the transport path length for both electrons and ions by using functionalized MWCNTs as structuring agent while maintaining its conductive nature.

## Results and Discussion

### Structural analysis

**Figure 2:**
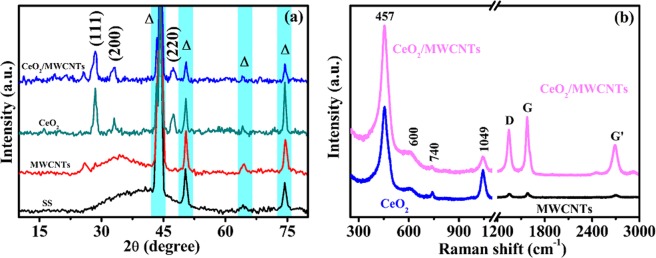
(**a**) XRD patterns of bare stainless steel (SS), MWCNTs, CeO_2_, and CeO_2_/MWCNTs thin films, (**b**) Raman spectra of CeO_2_/MWCNTs sample.

The structural investigation was performed by XRD and results are depicted in Fig. 2a. Both CeO_2_ and CeO_2_/MWCNTs films show three intense reflections at the 2θ values of 28.5°, 33.1° and 47.5° corresponding to the (111), (200) and (220) planes of cubic CeO_2_ (JCPDS card no. 81-0792). The relatively weak and broader reflection at 2θ = 25.8° observed in both the bare MWCNTs and the CeO_2_/MWCNTs thin films corresponds to amorphous carbon^10^.

Figure 2b shows the Raman spectra of the bare MWCNTs, CeO_2_, CeO_2_/MWCNTs samples. The strongest peak appearing at 457 cm^−1^ in the spectra of CeO_2_ and CeO_2_/MWCNTs corresponds to the Raman F_2g_ mode related to the stretching vibrations of oxygen^5^. The small hump around 600 cm^−1^ is assigned to the oxygen defective CeO_2_ layers^11^. Moreover, the typical internal vibration of nitrates are associated with the peaks arisen at about 740 and 1049 cm^−1^, respectively^12^.

The Raman spectrum of CeO_2_/MWCNTs also shows the characteristic D band (1346 cm^−1^) and G band (1578 cm^−1^), respectively arising from defects and the sp^2^ hybridized carbon atoms in MWCNTs walls^13^. The significant G′ (2D) peak (2691 cm^−1^) is observed due to phonon scattering in MWCNTs; indication of a dense uniform distribution of MWCNTs in the sample^14^. The peak intensities of D and G bands for CeO_2_/MWCNTs are enhanced as compared to bare MWCNTs due to the surface enhanced Raman scattering (SERS) effect associated with the CeO_2_ nanoparticles, which affect the local electromagnetic field^15^. The relative intensity ratio shows the information about graphitic and disordered carbon. An I_D_/I_G_ ratio of 0.88 for composite corresponds to the enhanced electronic conductivity of the electrode.

**Figure 3:**
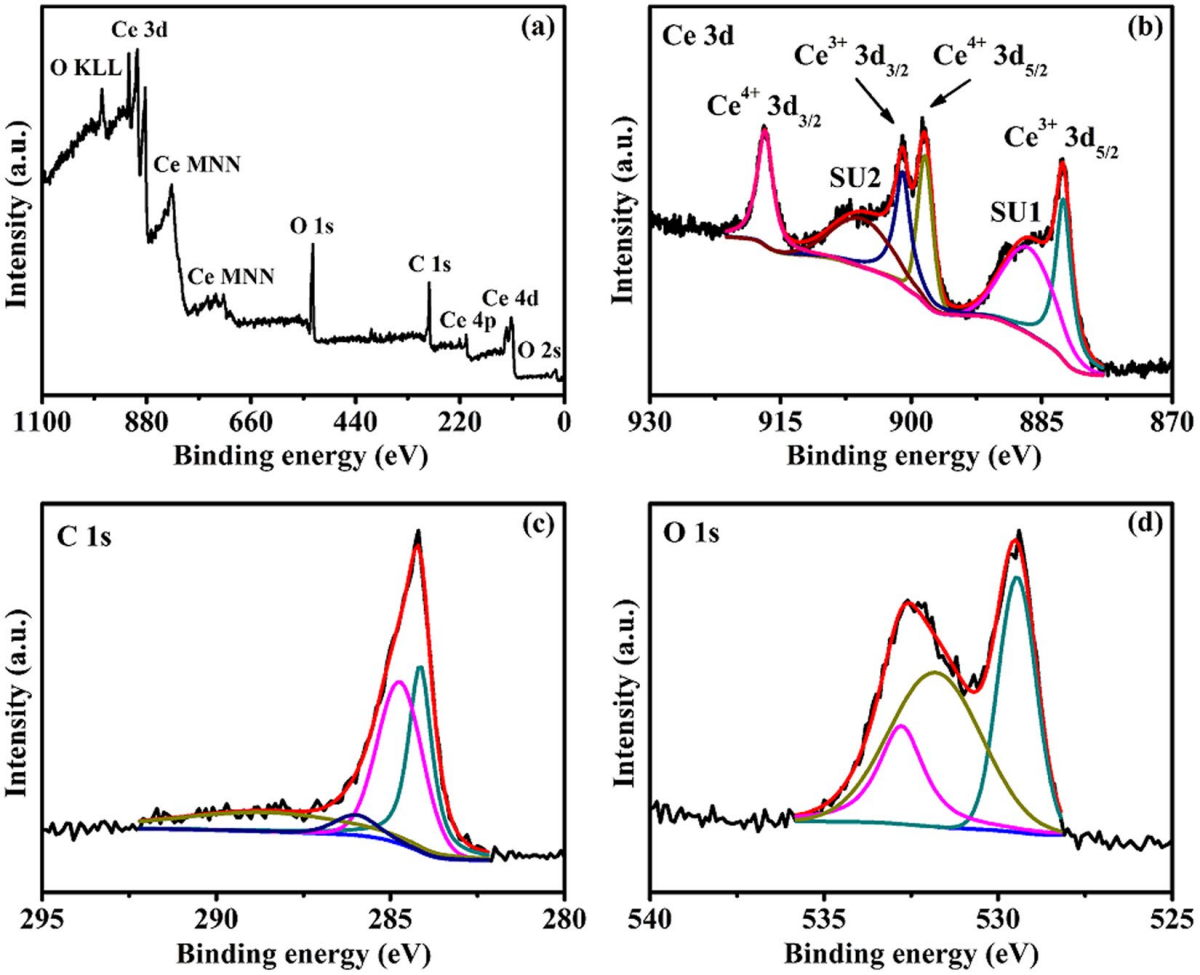
(**a**) XPS survey spectrum of CeO_2_/MWCNTs, (**b–d**) core level XPS spectra of Ce 3d, C 1s, and O 1s.

The oxidation states of constituent elements were analyzed by XPS analysis (Fig. 3a). To determine the peak position, the featured survey and core spectra were fitted by nonlinear least-square fitting (NLLSF) method associated with Gaussian–Lorentz distribution function after Shirley integrated background subtraction. The survey spectrum is similar with previous literatures^16,17^. KLL and MNN are the Auger groups for O and Ce, respectively^18^.

The main peaks such as Ce^4+^ 3d_3/2_ and Ce^4+^ 3d_5/2_ related Ce 3d core spectrum can be clearly shown at binding energies of 916.77 and 898.38 eV, respectively (Fig. 3b). Moreover, the Ce^3+^ 3d_3/2_ and Ce^3+^ 3d_5/2_ peaks are assigned at 900.97 and 882.50 eV, individually. Two additional ‘shake-up’ satellite lines specified by SU1 and SU2 are revealed at 905.63 and 886.49 eV, separately. This Ce 3d spectrum is well consistent with previously reported literatures^19^. The existence of Ce^3+^ is a consequence of oxygen vacancies which is superior in nanoparticles. Since, greater number of the atoms are on the surface, the surface atoms will reduce coordination because of reduced particle size^5^. The oxygen vacancies lead to the transformation between Ce^4+^ and Ce^3+^ which is the main factor for enhanced electrochemical reactions on electrode. It is clear that CeO_2_/MWCNTs composite has a good oxygen release and storage capacity through the reversible redox reactions between Ce^3+^ and Ce^4+^ under reducing and oxidizing conditions, respectively.

As shown in Fig. 3c, the peak of C 1s spectrum at 284.14 eV specifies the non-oxygenated C–C bonds whereas the other peaks are due to the oxygenated functional groups present in MWCNTs. The component peak at 284.72 eV is associated with the C atoms directly bonded to hydroxyl group (C–OH). The peak related to carbonyl group (C=O) is indicated at 285.98 eV and the peak at 288.57 eV is due to the carboxyl group (O=C–OH)^20^.

Three Gaussian components in O 1s spectrum (Fig. 3d) at 529.46, 531.80 and 532.78 eV correspond to the phenolic, carboxylate C–OH and C=O groups, respectively^21,22^. It is noticeable that the peak assigned at 531.80 eV indicates a greater contribution than from C–OH and C=O bonds. The analyzed results of O 1s spectrum clearly support the details defined by C 1s spectrum.

### Morphological analysis

**Figure 4:**
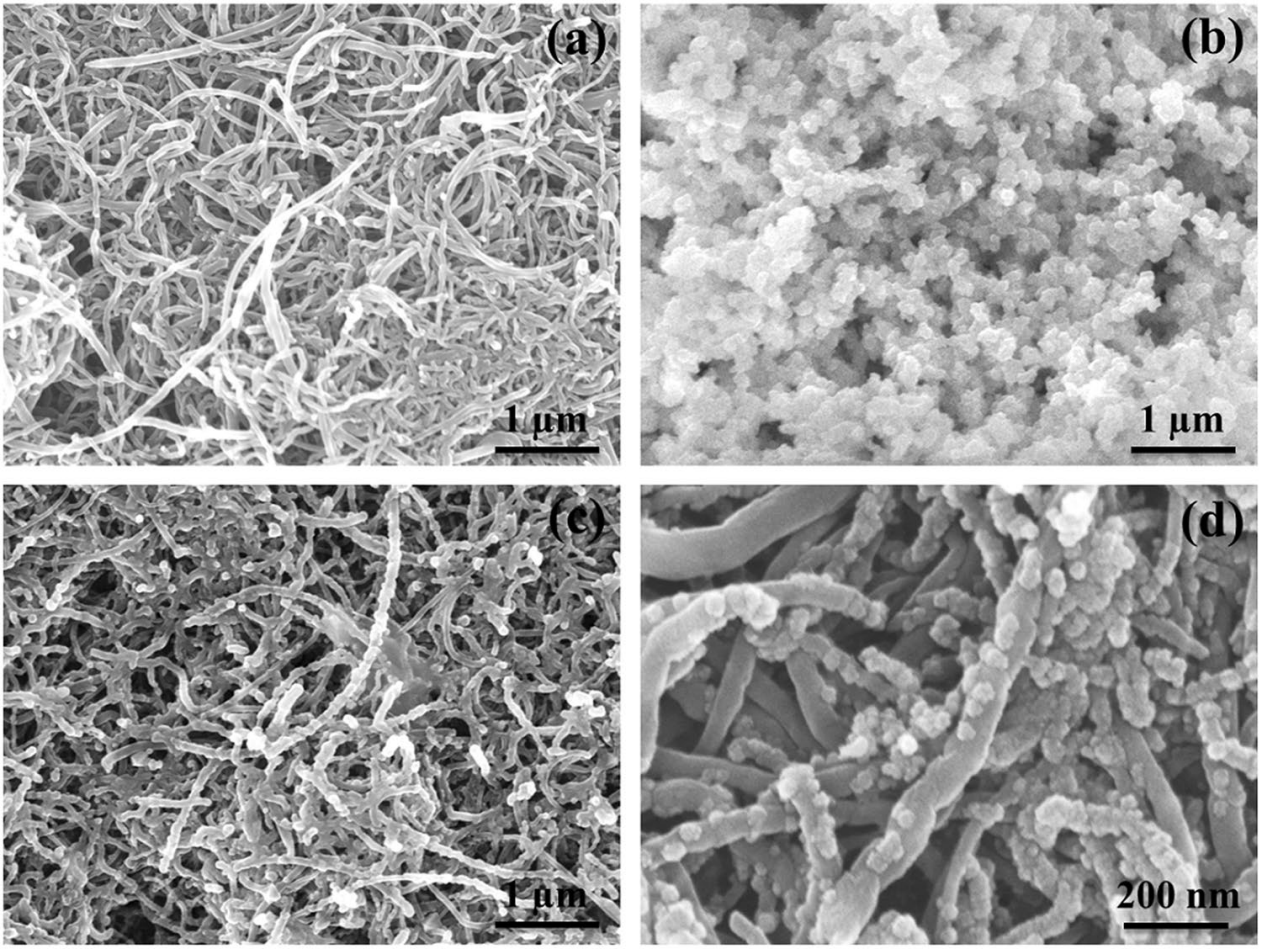
(**a**–**c**) FESEM images of as-prepared MWCNTs, CeO_2_, and CeO_2_/MWCNTs thin films on SS substrate at 1 μm magnification, (**d**) FESEM image of CeO_2_/MWCNTs at magnification of 200 nm.

As shown in Fig. 4a, the surface of the stainless steel (SS) substrate is well covered by a complex MWCNT network, while the hierarchical nanoparticle morphology of CeO_2_ onto SS substrate (as reference) offers plenty of electroactive surface to the electrolyte ions (see Fig. 4b). FESEM images of the CeO_2_/MWCNTs composite (Fig. 4c,d) reveal that the CeO_2_ nanoparticles are well encapsulated on the outer surface of MWCNTs. It is well noted that the CeO_2_/MWCNTs nanostructured material providing high surface area (Supplementary Information S1**)** with enough space not only opens well-established conductive network for electrolyte diffusion and electron transport but also offers sufficient electroactive sites for electrochemical redox reactions. The cross-linked and intertwisted network of the porous MWCNTs film greatly supports the conductive pathway between oxide layer and the metal substrate, and hence, significantly reduces the charge transfer resistance with increase in the utilization rate of the active materials and consequently, results in the outstanding capacitance value.

**Figure 5:**
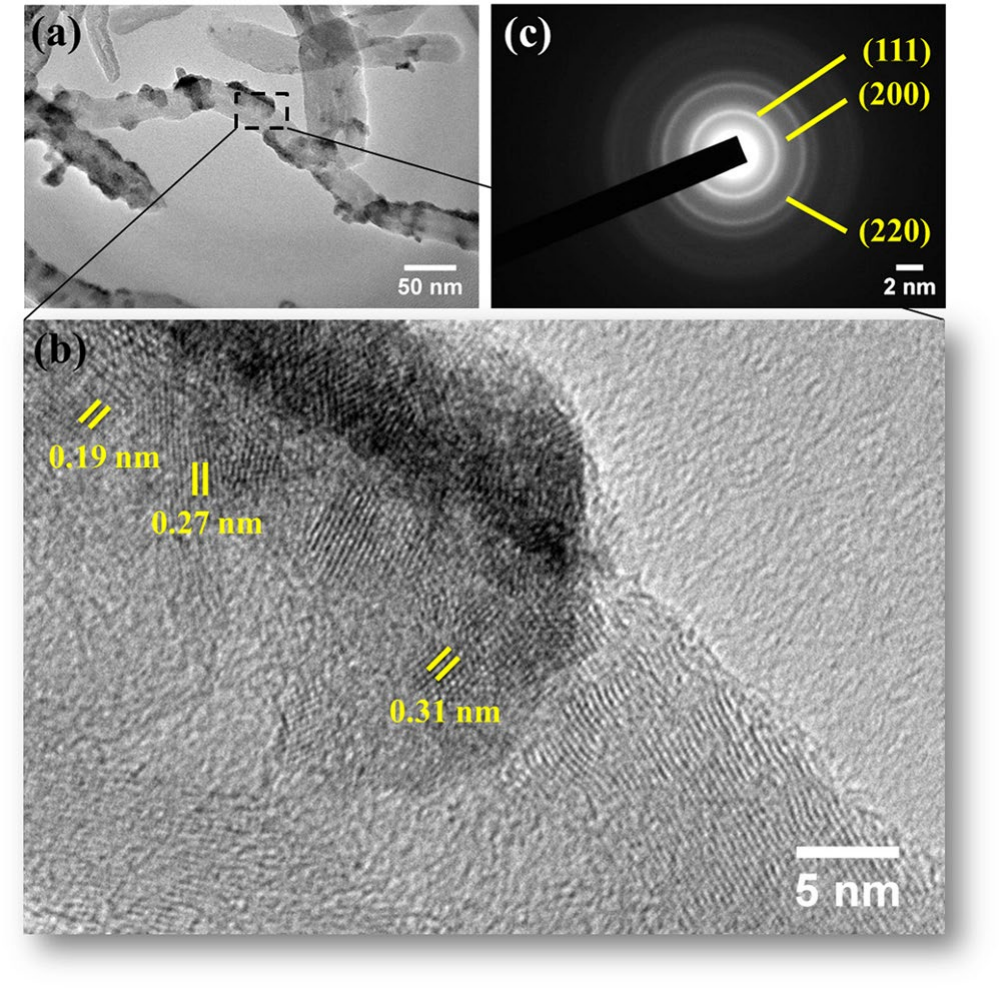
(**a,b**) HRTEM images of CeO_2_/MWCNTs at different magnifications, (**c**) corresponding SAED pattern.

The detailed structure is revealed by HRTEM images depicted in Fig. 5a,b. The images depict the anchoring of CeO_2_ onto MWCNTs to form nanocomposite which is in well agreement with the FESEM images, confirming the successful synthesis of the CeO_2_/MWCNTs composite with hierarchical structure. The anchoring of CeO_2_ with the surface attached carboxyl groups of the MWCNTs prevents agglomeration among CeO_2_ nanoparticles resulting in the higher dispersion^23^.

**Figure 6:**
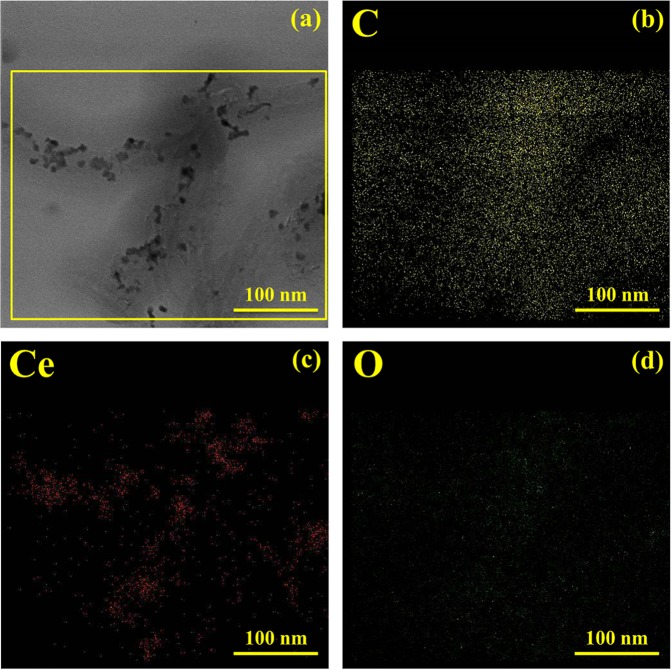
(**a–d**) EDS mapping analysis; the yellow, red, and green color represents carbon (**c**), cerium (Ce), and oxygen (O) elements, respectively.

Figure 5b displays the lattice fringes with interplanar spacing of 0.31, 0.27, and 0.19 nm, corresponding to the (111), (200), and (220) planes of CeO_2_ respectively. A set of concentric rings appeared in SAED pattern (Fig. 5c) denote the same (111), (200), and (220) planes of the cubic structured CeO_2_. Both these results are supportive to the data obtained through XRD studies. EDS elemental mapping analysis of elements C, Ce, and O (shown in Fig. 6b–d, respectively) from a selected area of the hybrid nanostructure (Fig. 6a) confirms the coating of CeO_2_ nanostructures on MWCNTs.

### Supercapacitor properties of the electrodes

**Figure 7:**
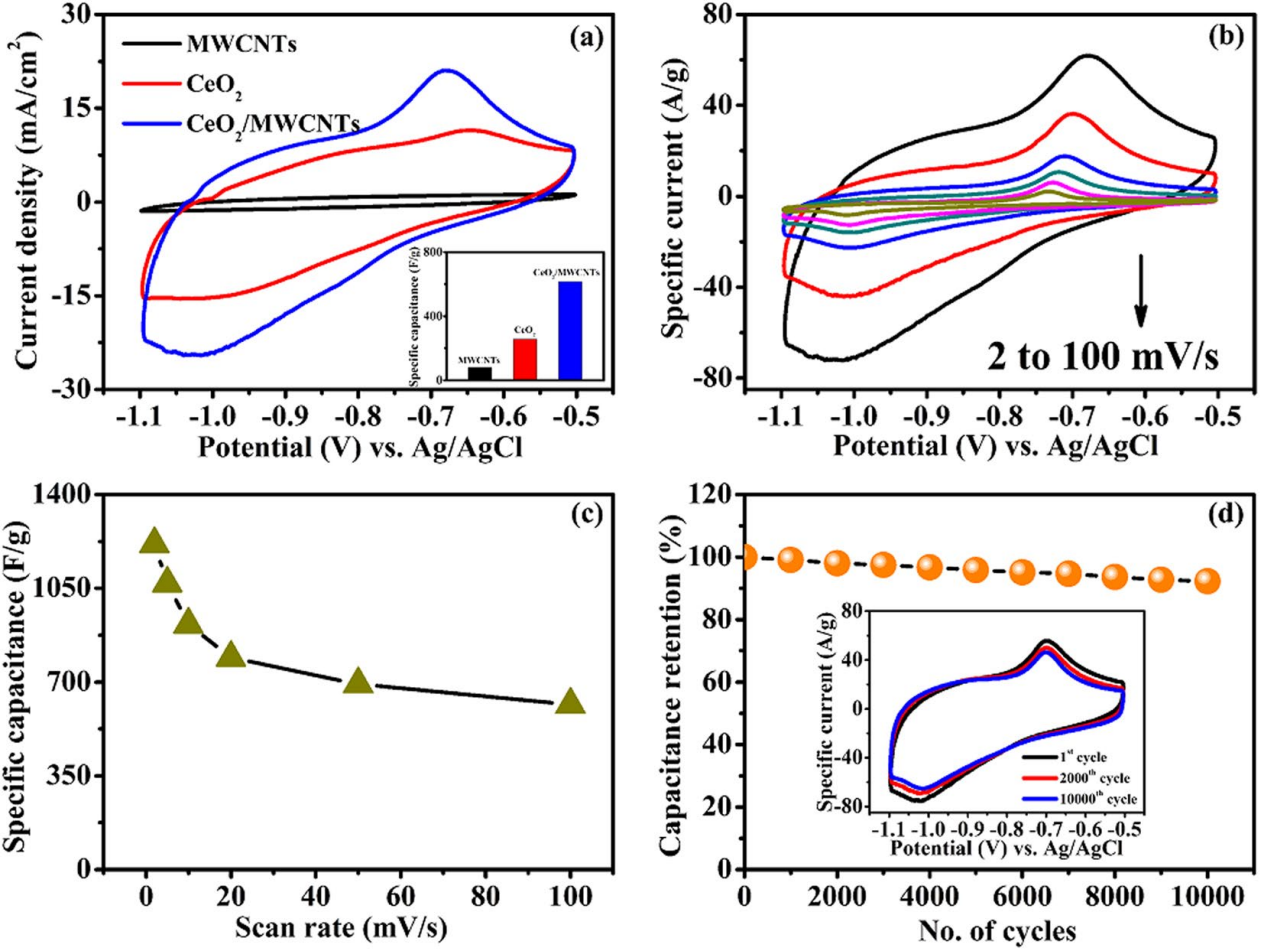
Electrochemical performances in 0.5 M NaOH electrolyte. (**a**) CV curves for MWCNTs, CeO_2_ and CeO_2_/MWCNTs electrodes at scan rate of 100 mV/s, inset shows specific capacitance of corresponding electrodes, (**b**) CV curves of CeO_2_/MWCNTs electrode at different scan rates ranging from 2 to 100 mV/s, (**c**) specific capacitance as a function of scan rate, (**d**) cycling stability at 10000 cycles, inset shows the CV curves for different cycle numbers at 100 mV/s scan rate.

The electrochemical properties of the as-grown hierarchical CeO_2_ nanostructures on the MWCNTs surface were explored at a scan rate of 100 mV/s in 0.5 M NaOH electrolyte within a potential window of −0.5 to −1.1 V (Supplementary Information S2). As reference, CeO_2_ and MWCNTs nanostructures were also assessed and shown in Fig. 7a. The CV curve of MWCNTs displays a distinct capacitive behavior which is consistent with previous reports^24^. Both the CeO_2_ and CeO_2_/MWCNTs composite electrodes exhibit well-defined oxidation-reduction peaks. Additionally, the CeO_2_/MWCNTs electrode exhibits the highest current responses, with approximately similar areas in the anodic and cathodic regions, resulting superior capacitance value (inset, Fig. 7a). The scan rate dependent CV curves are shown in Fig. 7b. The CeO_2_/MWCNTs electrode exhibits a much superior capacitance of 1215.7 F/g at scan rate of 2 mV/s (Fig. 7c). The origin of the peaks is associated to the following reversible redox reaction^25^:

1 $${{\rm{Ce}}}^{{\rm{IV}}}{{\rm{O}}}_{2}+{{\rm{OH}}}^{-}\rightleftharpoons {{\rm{Ce}}}^{{\rm{III}}}{{\rm{O}}}_{2}{\rm{OH}}+{{\rm{e}}}^{-}$$

The CV curve of CeO_2_/MWCNTs composite electrode principally retains faradaic peaks even at scan rates up to 100 mV/s, which is symptomatic of a fast charge transport, i.e., pseudocapacitance, in the electrode. Both the magnitudes of the current and the potential peak separation increase with the rise in scan rate, and the oxidation and reduction peaks shift towards more positive and negative values, respectively, mainly due to polarization and ohmic resistance appeared during the faradaic processes^26^.

The cyclic stability of the electrode was executed at 100 mV/s scan rate within the same potential window. The unperturbed CV curves even at 10000 cycles are depicted in the inset of Fig. 7d. Notably, the composite electrode exhibits very high capacitance retention, maintaining 92.3% of its initial capacitance at 10000 cycles (Fig. 7d). These remarkable results prove that CeO_2_/MWCNTs is chemically stable in NaOH electrolyte with very low degradation, constantly delivering power upon long-term cycling with good reversibility in spite of its pseudocapacitive behavior. In fact, MWCNTs supports the prevention of the degradation of electroactive material to electrolyte solution by strong synergy during electrochemical reaction process.

**Figure 8:**
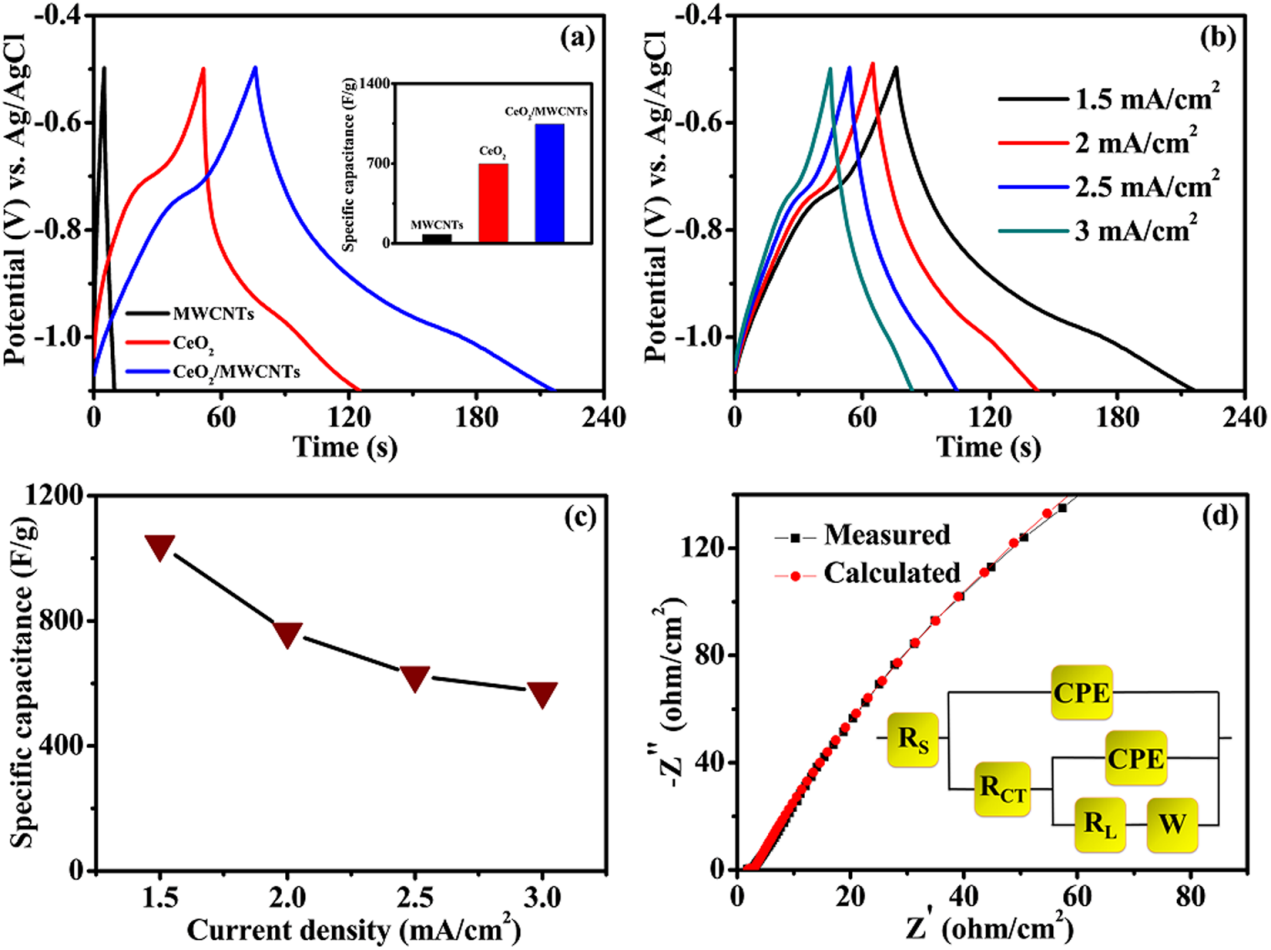
(**a**) CD curves of MWCNTs, CeO_2_ and CeO_2_/MWCNTs electrodes at current density of 1.5 mA/cm^2^; inset shows specific capacitance of respective electrodes, (**b**) CD curves at different current densities ranging from 1.5 to 3 mA/cm^2^, inset shows specific capacitance as a function of current density, (**d**) Nyquist plot of impedance from 100 mHz to 100 kHz, inset shows corresponding equivalent circuit.

The effect of the current density on the specific capacitance of MWCNTs, CeO_2_, and CeO_2_/MWCNTs electrodes was investigated by galvanostatic charge/discharge (CD) studies. Figure 8a demonstrates the CD curves of the supercapacitors at a CD current density of 1.5 mA/cm^2^. The MWCNTs electrode shows electric double layer capacitance (EDLC), exhibiting the typical triangular CD plot. However, both the CeO_2_ and the CeO_2_/MWCNTs electrodes show pseudocapacitive characteristics^27^. Moreover, the CeO_2_/MWCNTs composite electrode shows the greatest discharge time at a same current density of 1.5 mA/cm^2^, exhibiting greater capacitance as shown in inset of Fig. 8a. The discharge curve includes two stages in the voltage ranges between −0.5 to −0.8 and −0.8 to −1.1 V. The first stage, with a comparatively smaller duration is attributed to EDLC; whereas, the second stage, with a much longer discharge duration, involves the combination of EDLC and faradaic capacitance originating from MWCNTs and CeO_2_, respectively^28^. Furthermore, CD curves for composite were analyzed with different current densities ranging from 1.5 to 3 mA/cm^2^ and depicted in Fig. 8b. Utilizing both the charge storage mechanisms, the CeO_2_/MWCNTs electrode yields a maximum specific capacitance of 1044.2 F/g at a current density of 1.5 mA/cm^2^, decreasing to 573.5 F/g at 3 mA/cm^2^ (Fig. 8c). A little iR drop at the starting of every discharge curves is observed due to the presence of internal resistance^29^.

Non-intrusive and highly sensitive EIS measurements were performed in the 0.1 Hz to 100 kHz frequency range. As shown in Nyquist plots in Fig. 8d, a semicircle in the high frequency region is observed, resulting from the charge-transfer resistance (R_CT_) associated to the faradaic reactions. The equivalent series resistance (R_S_) constituted by the electrolyte resistance, the contact resistance between the active material and the current collector, and the intrinsic resistance of the electro-active material is obtained from the high frequency intersect of the impedance curve^30^. The electrode shows low values of R_S_ (1.87 Ω/cm^2^) and R_CT_ (1.06 Ω/cm^2^), these obtained low values are of great importance as they affect the power and energy performance, but also reduce undesired heat dissipation throughout the charge–discharge processes^31^. The reason behind the optimal behavior is that the MWCNTs can effectively transport the current to and from the active material (Supplementary Information S3**)**. Additionally, they prevent the agglomeration of the CeO_2_ nanoparticles, facilitating the contact between the active material (CeO_2_) and the electrolyte, which in turns lead to the improved utilization of the active material (i.e., to high capacitance). Moreover, the fitted equivalent circuit of the Nyquist plot is presented in the inset of Fig. 8d. The constant phase element (CPE) represents the double layer capacitance occurring at the interface between the electroactive material and the electrolyte^32^. The impedance due to the diffusion of OH^−^ ions within pores of the CeO_2_ electrode is represented by the Warburg element, and is dependent upon the frequency whereas R_L_ represents the leakage resistance during the electrochemical activities^33^.

**Figure 9:**
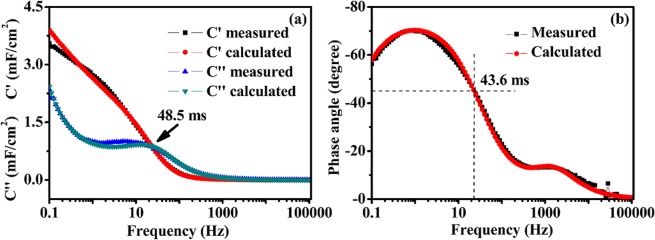
(**a**) Real and imaginary capacitance vs. frequency plot, (**b**) Bode plot.

Figure 9a shows the Bode plots of the real (C′) and imaginary (C″) components of the capacitance against the frequency. The variation of C″ with the frequency shows a maxima at the characteristic frequency (f_0_ = 20.6 Hz), defining the relaxation time as ${{\rm{\tau }}}_{0}=\frac{1}{{{\rm{f}}}_{0}}$. The time ${{\rm{\tau }}}_{0}$ is a measure of the rate capability of the supercapacitor as it is normally associated with the swiftness of the capacitive discharge. The relaxation time is the minimum time required to deliver the stored energy with an efficiency greater than 50% of its maximum value and redirects the shift among the resistive and capacitive activities of the supercapacitor^34^. The small relaxation time constant of 48.5 ms suggests the excellent charge-discharge rate performance of the electrode material. The experimental impedance data has been converted to imaginary capacitance (C″) using the following equation.

2 $${\rm{C}}^{\prime\prime} =\frac{{\rm{Z}}^{\prime} }{2{\rm{\pi }}f\,{|{\rm{Z}}|}^{{\rm{2}}}}$$

The phase angle is −77° (close to 90°) as exhibited in the Bode plot (Fig. 9b), suggesting excellent capacitive response^35^. As capacitive and resistive impedances are identical at phase angle of −45°, relaxation time constant can be estimated by ${{\rm{\tau }}}_{0}=\frac{1}{{{\rm{f}}}_{0}}$ at that particular phase and found to be 43.6 ms, consistent with the results obtained from the previous result.

### Electrochemical performance of flexible symmetric solid-state supercapacitor (FSSC)

**Figure 10:**
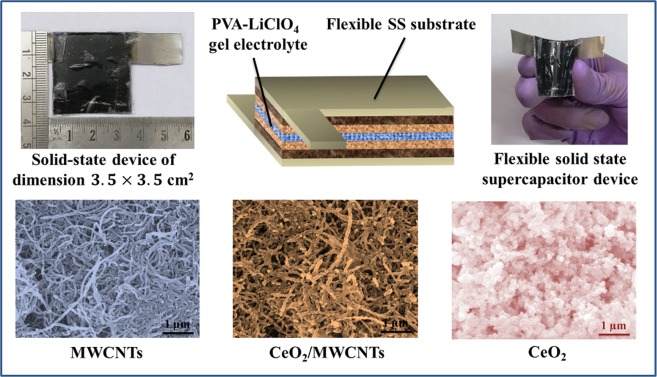
Schematic representation of the fabricated FSSC device based on CeO_2_/MWCNTs electrode and PVA-LiClO_4_ gel electrolyte.

Recent literatures suggest that the measurements performed on two-electrode cells are more effective than those made on three-electrode cells to ascertain the performance of the electrode materials (including the synthesis route) and electrolytes for commercial purposes. Considering the fast ionic transport property and high capacitance of the CeO_2_/MWCNTs composite, a symmetric solid-state supercapacitor device of dimension 3.5 × 3.5 cm^2^ was assembled using PVA-LiClO_4_ gel as solid-state electrolyte and separator. As shown in Fig. 10, the resulting device could be bent and twisted easily, which combines additional advantage compared to other SCs.

**Figure 11:**
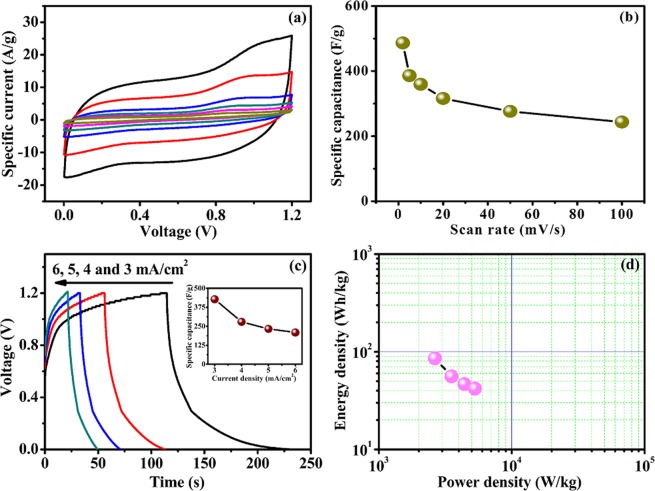
Electrochemical performance of FSSC device with PVA-LiClO_4_ gel electrolyte. (**a**) CV curves at different scan rates ranging from 100 to 2 mV/s with voltage window of 1.2 V, (**b**) specific capacitance as a function of scan rate, (**c**) CD curves at different current densities ranging from 3 to 6 mA/cm^2^, inset shows specific capacitance as a function of current density, (**d**) Ragone plot.

The fabricated symmetric SC showed the characteristic pseudocapacitive behavior exhibiting oxidation and reduction peaks in the potential up to 1.2 V (Fig. 11a). The scan rate dependent CV curves of the FSSC consist of well-defined and almost symmetric peaks as a result of the reversible redox reactions of CeO_2_ involving Li^+^ insertion/release. Till date, many reports are available on high performance solid-state symmetric devices based on excellent electroactive materials. Chen *et al*. have constructed a symmetric device by using hybrid SWCNTs/RuO_2_ electrodes with a specific capacitance of 138 F/g^36^. The specific capacitance of 159.6 F/g was reported by Hou *et al*. for the symmetric device using layered ZnS/CNTs composite^37^. Zhao *et al*. improved electrochemical properties by porous highly flexible and conductive cellulose-Mediated PEDOT:PSS/MWCNT composite assembly that allowed for an enhanced specific capacitance of 380 F/g^2^. In present case, the specific capacitance of the device calculated on the basis of CV curve is 486.5 F/g at scan rate of 2 mV/s in spite of having a total mass loading of 8.33 mg as depicted in Fig. 11b. The CV curves maintain its original shape with the inclusion of large potential window of 1.2 V even at high scan rate (100 mV/s). Asymmetric device takes account of both the advantages of positive and negative electrodes, and hence exhibiting excellent electrochemical behavior than the symmetric devices. But, our fabricated symmetric device shows greater capacitance than many asymmetric devices reported by Liu *et al*. for carbon aerogel//Co_3_O_4_ (57.4 F/g)^38^, Wang *et al*. for NiCo-LDH//carbon nanorods (147.6 F/g)^39^, Kong *et al*. for NiCo_2_O_4_@PPy//activated carbon (AC) (165.4 F/g)^40^, Choi *et al*. for graphene(IL-CMG)//RuO_2_–IL-CMG (175 F/g)^41^, and Yan *et al*. for Ni–Co@Ni–Co LDH//carbon fibers (319 F/g)^42^.

The charge-discharge (CD) has also been analyzed with different current densities varying from 3 to 6 mA/cm^2^ and shown in Fig. 11c. The total specific capacitance of the device obtained as 428.3 F/g at a current density of 3 mA/cm^2^ is quite higher than other solid-state devices reported recently too (inset, Fig. 11c). Though various resistive factors are involved during device fabrication, the discharge curves show small iR drop by referring internal resistance. The Ragone plot relating the energy and power densities is shown in Fig. 11d. The device shows a maximum specific energy of 85.7 Wh/kg with the power density of 2.6 kW/kg. Moreover, the device can deliver energy density of 41.9 Wh/kg with the upsurge in power density of 5.3 kW/kg. The energy density of present device is substantially higher than those of recently reported all-solid-state symmetric SCs with electrode materials such as MoS_2_/carbon cloth (5.42 Wh/kg)^43^, GO/PPy (15.1 Wh/kg)^44^, SWCNTs/RuO_2_ (18.8 Wh/kg)^36^, rGO-PEDOT/PSS (2.83 Wh/kg)^45^, MWCNTs (3.5 Wh/kg)^46^, N-doped cotton-derived carbon frameworks (NCCF)-rGO (20 Wh/kg)^47^, MoSe_2_ (36.2 Wh/kg)^48^, PEDOT:PSS/MWCNT (13.2 Wh/kg)^2^, ZnS/CNTs (22.3 Wh/kg)^37^, ZnCo_2_O_4_/rGO (11.44 Wh/kg)^49^, Pt/n-CNT@PANI (30.22 Wh/kg)^50^, waste paper fibers-RGO–MnO_2_ (19.6 Wh/kg)^51^, and porous carbon (7.22 Wh/kg)^52^. Due to current collector-free feature, present ultrathin device showed remarkable energy density, a value considerably higher than even solid-state asymmetric devices, such as CNT/polyaniline//CNT/MnO_2_/GR (24.8 Wh/kg)^53^, carbon aerogel//Co_3_O_4_ (17.9 Wh/kg)^38^, graphene(IL-CMG)//RuO_2_–IL-CMG (19.7 Wh/kg)^41^, TiN@GNSs//Fe_2_N@GNSs (15.4 Wh/kg)^54^, NiCo_2_O_4_@PPy//activated carbon (AC) (58.8 Wh/kg)^40^, NiCo_2_O_4_/CC//porous graphene papers (PGP) (60.9 Wh/kg)^55^, γ-MnS//eggplant derived AC (EDAC) (37.6 Wh/kg)^56^, CoS//AC (5.3 Wh/kg)^57^, CoMoO_4_@NiMoO_4_•xH_2_O//Fe_2_O_3_ (41.8 Wh/kg)^58^, CuS/3D graphene//3D graphene (5 Wh/kg)^59^, MnO_2_@PANI//3D graphene foam (GF) (37 Wh/kg)^60^, NiCo_2_S_4_/polyaniline//AC (54.06 Wh/kg)^61^, NiCo-LDH//carbon nanorods (59.2 Wh/kg)^39^, Ni(OH)_2_/RGO/Ni//RGO aerogel/Ni (24.5 Wh/kg)^62^, and rGO/CoAl-LDH//rGO (22.6 Wh/kg)^63^.

**Figure 12:**
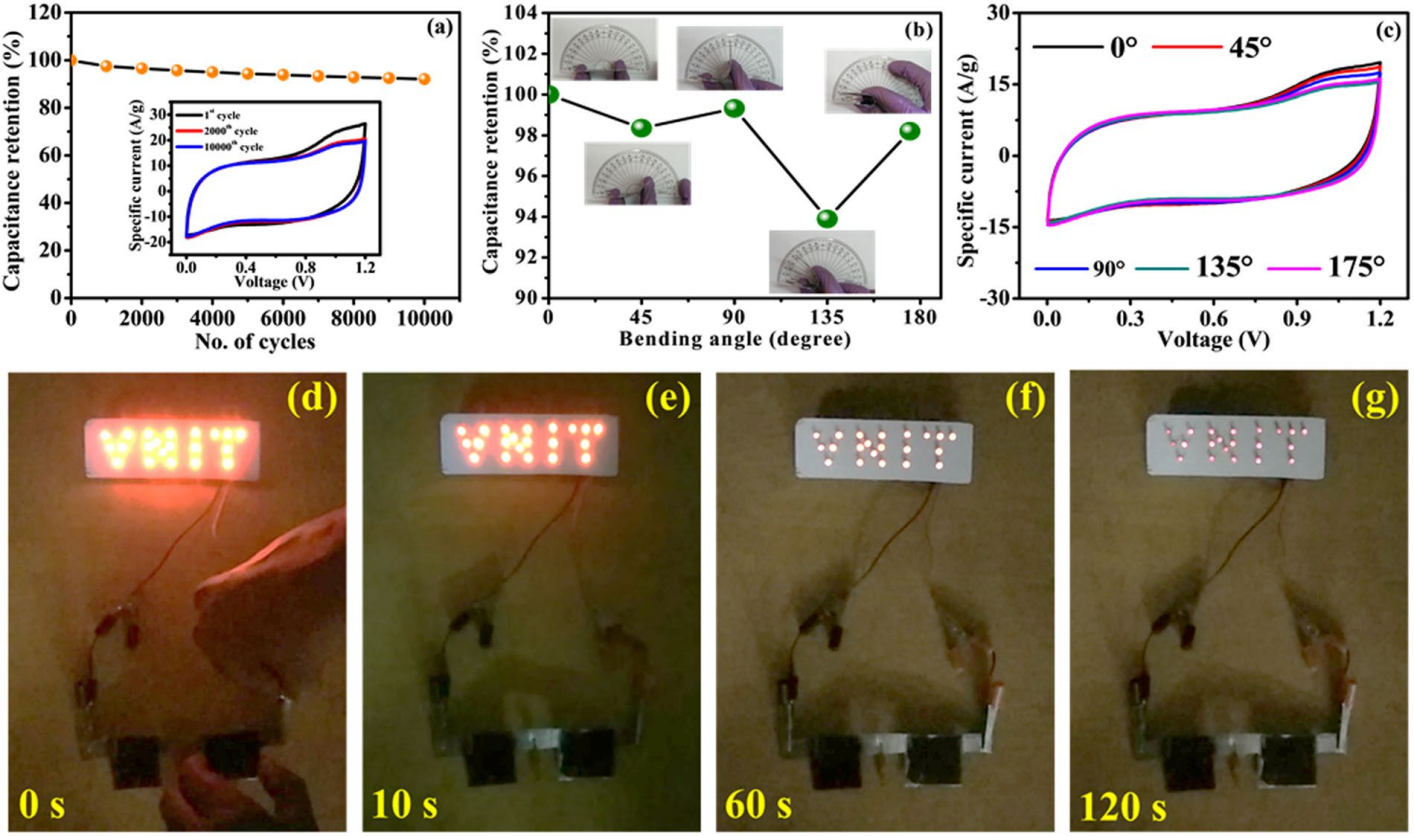
(**a**) Cycling stability at 10000 cycles with 100 mV/s scan rate, inset shows CV curves for different cycle numbers at scan rate of 100 mV/s, (**b**) capacitance retention at different bending angles, (**c**) CV curves with different bending angles at scan rate of 100 mV/s, (**d–g**) actual demonstration of FSSC device discharging through ‘VNIT’ panel consisting of 21 red LEDs for 0, 10, 60 and 120 s respectively.

Towards stability check, CV was repeated 10000 times for FSSC device and results are shown in Fig. 12a. There was a quick drop of specific capacitance for first 1000 cycles and stabilized after 2000 cycles. At 10000 cycles, it shows an excellent retention of 92.1% which is higher than recently reported solid-state devices (Supplementary Information S4) and strongly favors the commercial use of FSSC device. Continuously, the performance durability of the FSSC device was further tested under harsh mechanical conditions through bending states by bending the assembled symmetric device at various degrees from 0 to 175° where almost no loss in the capacitance (98.2% retention at 175°) is observed Fig. 11b; suggesting its superior mechanical stability under stress environment. This superior performance is attributed to the excellent adhesion of gel electrolyte, good mechanical robustness, electrical conductivity, intimate interfacial contact among multiple components, and well adherence of MWCNTs to the SS substrate. The overlapping of CV curves of all bending angles shows the high rate capability of the electrode material (Fig. 12c).

As practical demo, a series combination of two SC devices was assembled and charged with 2.4 V. The system can easily lighten up a ‘VNIT’ panel consisted of 21 red LEDs with luminous intensity for 120 s duration (Supplementary video) as depicted in Fig. 12d–g which explores the potential ability of the device. The successful attempt to drive commercial LEDs shows that our device has the opportunity to be applied in energy storage and portable/flexible electronics.

As compared with recently reported solid-state devices, present assembled symmetric device shows superior supercapacitive characteristics including excellent energy and power densities which can be described in the following aspects: (i) unique morphology by the hybridization of CeO_2_ nanoparticles and MWCNTs nanostructures with richer specific surface and porosity, enabling short electron transport paths and high rate of charge propagation which overall improves the electrochemical performance, (ii) use of MWCNTs is not only to encapsulate CeO_2_ nanostructure with strong synergy but to enhance the conductivity and stability of the prepared composite too, and (iii) combining two symmetric CeO_2_/MWCNTs electrodes using PVA-LiClO_4_ gel extends the voltage window up to 1.2 V, ensuing remarkable enhancement in energy density of the device and empower the device to one step closer to hands-on application.

## Conclusions

Hierarchical CeO_2_ nanostructure was successfully anchored onto outer surface of MWCNTs using facile chemical method. Supercapacitor based on hybrid CeO_2_/MWCNTs nanostructured electrode displays an enhanced capacitive performance in terms of specific capacitance of 1215.7 F/g, cyclic stability of 92.3% at 10000 cycles and low vaues of resistive factors (R_S_ = 1.87 Ω/cm^2^ and R_CT_ = 1.06 Ω/cm^2^). The assembled device impressively shows excellent supercapacitive performance with superb electrochemical and mechanical stability. The durability operation was achieved successfully with a wide cell voltage of 1.2 V, giving rise to energy and power densities. Well-integrated interface between the electrodes and the electrolyte enables fast charge storage/release processes at high rates and good cycling performance over 10000 cycles even under harsh mechanical (bent) conditions. These rationally designed symmetric SCs represent a promising pathway to build-up flexible energy storage devices with high-performance to drive wearable and stretchable electronic devices for advance applications.

## Experimental

### Fabrication of electrodes and devices

Previously reported synthesis procedure to coat MWCNTs onto SS substrate has been adopted^64,65^. Briefly, 95% pure MWCNTs (length = 5–15 μm and Outward diameter = 20–40 nm) procured from Monad Nanotech Pvt. Ltd. (Maharashtra, India) were refluxed using H_2_O_2_ at 90 °C for 48 h in order to anchor oxygenated functional groups by removing amorphous carbon derivatives. The residue was rinsed repeatedly in double distilled water (DDW) followed by drying at 60 °C for 12 h. To obtain well-dispersed solution of MWCNTs, the product was processed through ultra-sonication in 1 wt% Triton X-100 and DDW with the ratio of Tx-100:DDW equal to 0.01. The two-step mechanical process involving immersion of SS substrate into the dispersed MWCNTs solution and dehydration under IR lamp yields uniform and well adherent coating of MWCNTs on SS substrate.

The simple chemical bath deposition (CBD) method was employed to deposit cerium oxide over the pre-coated MWCNTs (Supplementary Information S5). In brief, cerium (III) nitrate (Ce(NO_3_)_3_, 6H_2_O) was used as cationic solution, while hydrogen peroxide (H_2_O_2_, 30%) was used as anionic precursor during the process. The precursor solution was prepared by dissolving 0.04 M Ce(NO_3_)_3_ in 50 ml DDW under constant stirring to get uniform distribution. Furthermore, 2.5 mL H_2_O_2_ was added to the prepared solution under vigorous stirring. After, the MWCNTs coated substrate was immersed in the bath kept at a constant temperature of 60 °C. After 1 h, the yellowish cerium oxide deposited MWCNTs substrate was taken out from the bath and rinsed several times in DDW and dried under infrared (IR) radiation. The as-prepared film was air annealed at 200 °C to remove extra hydroxide. The synthesis involves two steps. Firstly, ${{\rm{Ce}}}^{3+}$ ions in association with H_2_O_2_ form complex ${\rm{Ce}}{{({\rm{OH}})}_{2}}^{2+}$ ions as:

3 $$2{{\rm{Ce}}}^{3+}+2{{\rm{OH}}}^{-}+{{\rm{H}}}_{2}{{\rm{O}}}_{2}\to 2{\rm{Ce}}{{({\rm{OH}})}_{2}}^{2+}$$

In the second reaction step, ${\rm{Ce}}{{({\rm{OH}})}_{2}}^{2+}$ yields CeO_2_ as thin film form on SS substrate.

4 $${\rm{Ce}}{{({\rm{OH}})}_{2}}^{2+}\to {{\rm{CeO}}}_{2}+2{{\rm{H}}}_{2}{\rm{O}}$$

The non-oxidized hydroxide originating from reaction, was eliminated via the annealing process as follows:

5 $${{\rm{Ce}}}^{3+}+3{{\rm{OH}}}^{-}\to {\rm{Ce}}{({\rm{OH}})}_{3}$$

6 $${\rm{Ce}}{({\rm{OH}})}_{3}\mathop{\to }\limits^{200\,^\circ {\rm{C}}}{{\rm{CeO}}}_{2}+{{\rm{H}}}_{3}{{\rm{O}}}^{+}+{{\rm{e}}}^{-}$$

Further, the PVA-LiClO_4_ gel electrolyte was obtained by adding 6 g of LiClO_4_ and 6 g of polyvinyl alcohol (PVA) powder into 60 ml of DDW^66^. The mixture was heated at 90 °C under stirring until the solution became clear and viscous. Then the composite electrode on the flexible SS substrate was coated with a thin layer of the prepared PVA-LiClO_4_ gel electrolyte followed by evaporation of the excess water. When the gel electrolyte got solidified, the two electrodes were sandwiched and packaged to assemble flexible symmetric supercapacitor (FSSC) device.

### Characterizations

XRD was performed by Bruker AXS D8 Advance diffractometer using Cu K_α_ X-ray source. Raman studies were performed with LabRAM HR, 532 nm laser excitation. Energy-dispersive X-ray spectroscopy (EDX) was acquired using a JSM-7610F analyzer connected with a scanning electron microscope (FESEM, JEOL JSM-7610F). The detailed morphological study was performed by high-resolution transmission electron microscopy (HRTEM) using JEOL 2100 with LaB_6_ source. XPS measurements were carried out in a PHI 5000 VersaProbe II (ULVAC INC, Japan) photoelectron spectrometer under ultrahigh vacuum (UHV) below 5 × 10^−10^ Torr. XPS system is equipped with the Al K_α_ X-ray monochromator operated with an anode power of 350 W and the sample surface normal was oriented at 45° to both the X-ray source and photoelectron spectrometer.

Electrochemical characteristics of the as-obtained films were studied on PARSTAT 4000 electrochemical workstation (Princeton Applied Research, USA) using cyclic voltammetry (CV), charge-discharge (CD) and electrochemical impedance test on three-electrode cells. In these, the composite electrode acts as the working electrode, Pt wire as the counter electrode and a saturated Ag/AgCl electrode as the reference electrode.

## Supplementary information

Supplementary information

Supplementary video
