# Supplementary material for: Novel chemical route for CeO2/MWCNTs composite towards highly bendable solid-state supercapacitor device
Source: Sci Rep. 2019 Apr 10;9:5892. doi: 10.1038/s41598-019-42301-y (PMC6458112; doi:10.1038/s41598-019-42301-y)
Supplement: Supplementary file 1 — Supplementary information [file 41598_2019_42301_MOESM1_ESM.docx]

**Supplementary information for:**

**Novel chemical route for CeO_2_/MWCNTs composite towards highly bendable solid-state supercapacitor device**

**Bidhan Pandit^1^, Babasaheb R. Sankapal^1^*, Pankaj M. Koinkar^2^****

*^1^Nano Materials and Device Laboratory, Department of Physics, Visvesvaraya National Institute of Technology, South Ambazari Road, Nagpur-440010, Maharashtra, India.*

*^2^Department of Optical Science, Tokushima University, 2-1 Minamijosanjima Cho, Tokushima-7708506, Japan.*

**CORRESPONDING AUTHOR FOOTNOTE**

**Babasaheb R. Sankapal and Pankaj M. Koinkar**

E-mail: [brsankapal@phy.vnit.ac.in](mailto:brsankapal@phy.vnit.ac.in); [brsankapal@gmail.com](mailto:brsankapal@gmail.com) (B. R. Sankapal)

Tel.: +91 (712) 2801170; Fax No.:- +91 (712) 2223230

E-mail: koinkar@tokushima-u.ac.jp (P. M. Koinkar)

Tel.: +81 886569563, Fax No.: +81 886569563

**S1. BET surface area**

The BET analysis with nitrogen adsorption-desorption isotherms is shown in **Figure S1**. The CeO_2_/MWCNTs nanostructure shows BET surface area of 139.04 m^2^/g which is much higher than MWCNTs (42 m^2^/g). So the typical nanostructured morphology of CeO_2_ onto MWCNTs improves the surface area of composite electrode, resulting in enhanced electrochemical performance.


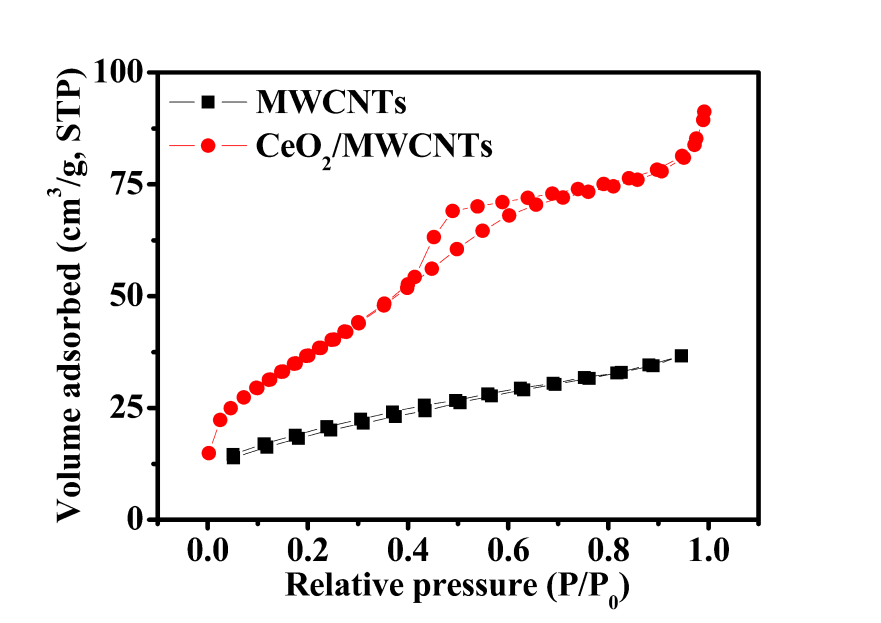


Figure S1: Nitrogen adsorption-desorption isotherms of MWCNTs and CeO_2_/MWCNTs.

**S2. Electrochemical characterizations**

Specific capacitance[^1^](#_ENREF_1) from the CV curves was calculated with the help of following formula:

$$C_{s}=\frac{1}{mv\Delta V}\int_{V_{i}}^{V_{f}} I\left( V \right)\mathrm{dV} \left( 1 \right)$$

where, ‘$C_{s}$’ signifies the specific capacitance (F/g), ‘$m$’ indicates mass (g) deposited on SS substrate, ‘$v$’ specifies scan rate (V), ‘$\Delta V$’ is an functional potential frame and ‘$\int_{V_{i}}^{V_{f}} I\left( V \right)\mathrm{dV}$’ represents area under the CV curve.

Specific capacitance ($C_{s}$), specific energy (E) in Wh/kg and specific power (P) in W/kg from Galvanostatic charge-discharge can be calculated using following equations:

$$C_{s}= \frac{I\Delta t}{m\Delta V} (2)$$

$$E=\frac{1}{2}\left[ \frac{C_{s}{\Delta V}^{2}}{3.6} \right] (3)$$

$$P=\frac{3600\times E}{\Delta t} \left( 4 \right)$$

where, ‘$I$’ represents current intensity and ‘$\Delta t$’ denotes discharge time (t) of the experimental charge-discharge curve.

**S3. Charge transfer resistance comparison**

**Figure S3a** shows the Nyquist plots of MWCNTs and CeO_2_/MWCNTs electrodes. The two electrodes show almost same equivalent series resistance (R_S_). The MWCNTs electrode exhibits higher charge transfer resistance (R_CT_) as 10.9 Ω/cm^2^ but reduces to 1.06 Ω/cm^2^ for the composite with CeO_2_. The presence of MWCNTs minimizes the internal resistance of CeO_2_/MWCNT composite by providing more and more conductive pathways for electrolyte ions.[^2^](#_ENREF_2)

Table S3: EIS data of CeO_2_/MWCNTs before and after stability studies.

|  | R_S_ (Ω/cm^2^) | R_CT_ (Ω/cm^2^) |
| --- | --- | --- |
| Before stability | 1.87 | 1.06 |
| After stability | 3.42 | 1.51 |

Furthermore, the Nyquist plot before and after stability studies are also analyzed and shown in **Figure S3b**. All the R_S_ and R_CT_ values are summarized in **Table S3**. The slight increase of R_S_ and R_CT_ values is due to slowdown of electrochemical activities due to the less penetration of electrolyte ions through the electrode surface with long-term cycles.[^3^](#_ENREF_3)^,^[^4^](#_ENREF_4)


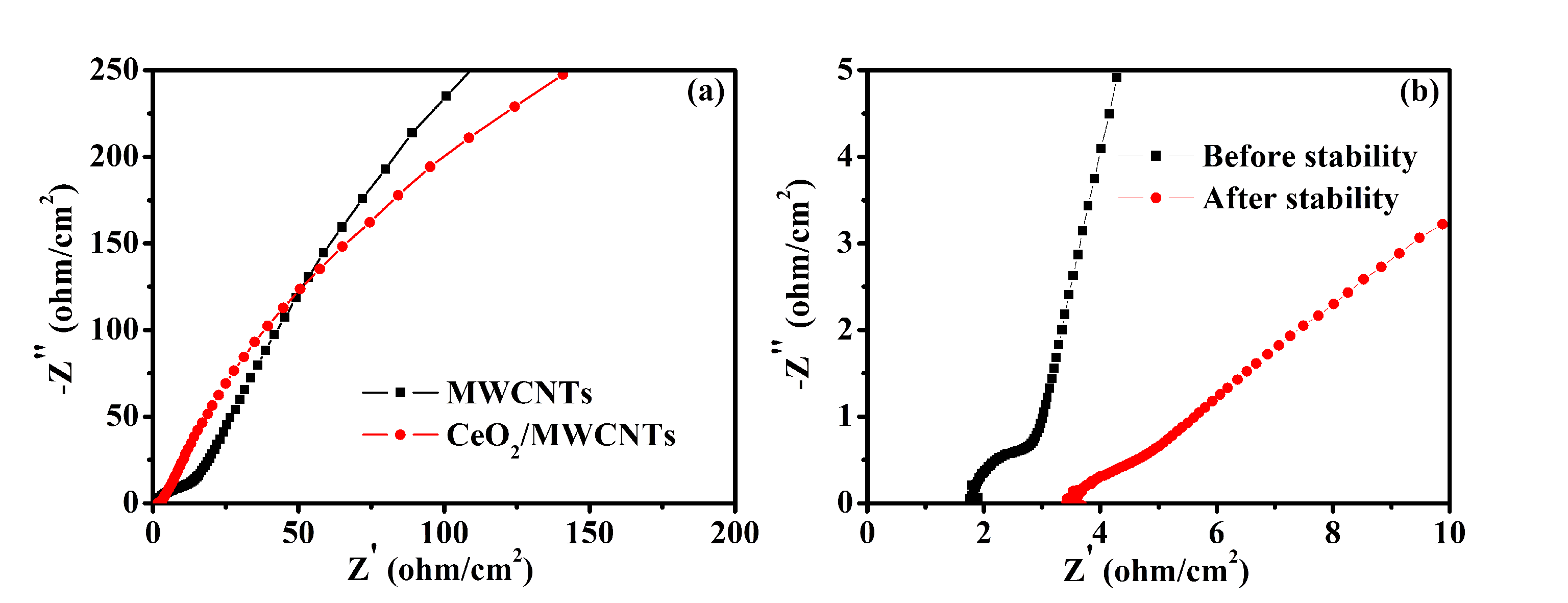


Figure S3: (a) Nyquist plot of MWCNTs and CeO_2_/MWCNTs electrodes, (b) Nyquist plot before and after stability studies of CeO_2_/MWCNTs electrode.

**S4. Performance of solid-state devices**

Summarized performance of FSSC device compared to recently reported solid-state supercapacitor devices

| Electrode materials | Device remarks | Electrolyte | Voltage window (V) | Specific capacitance (F/g) | Maximum Specific energy (Wh/kg) | Maximum  Specific power  (kW/kg) | Cyclic stability | | Ref. |
| --- | --- | --- | --- | --- | --- | --- | --- | --- | --- |
|  |  |  |  |  |  |  | Retention  (%) | Cycles |  |
| PEDOT:PSS/ MWCNT | Symmetric | PVA/KOH | 1 | 380 | 13.2 | 4.99 | 90 | 1000 | [^5^](#_ENREF_5) |
| SWCNTs/RuO_2_ | Symmetric | H_3_PO_4_/ PVA | 1 | 138 | 18.8 | 96 | - | - | [^6^](#_ENREF_6) |
| ZnS/CNTs | Symmetric | PVA–KOH | 1 | 159.6 | 22.3 | 5 | 91.8 | 3000 | [^7^](#_ENREF_7) |
| MoS_2_/carbon cloth | Symmetric | LiCl-PVA | 1.6 | 368 | 5.42 | 0.128 | 96.5 | 5000 | [^8^](#_ENREF_8) |
| GO/PPy | Symmetric | LiCl/PVA | 0.8 | 347.8 mF/cm^2^ | 15.1 | 4 | 86 | 2000 | [^9^](#_ENREF_9) |
| rGO-PEDOT/PSS | Symmetric | PVA/ H_3_PO_4_ | 1 | 448 mF/cm^2^ | 2.83 | 3.5895 | 95 | 10000 | [^10^](#_ENREF_10) |
| MWCNTs | Symmetric | PVA/ H_3_PO_4_ | 1 | 26.8 F/g | 3.5 | 28.1 | 92 | 5000 | [^11^](#_ENREF_11) |
| N-doped cotton-derived carbon frameworks (NCCF)-rGO | Symmetric | PVA/KOH | 1 | 200 | 20 | 4 | 94 | 10000 | [^12^](#_ENREF_12) |
| MoSe_2_ | Symmetric | PVA/KOH | 1.4 | 133 | 36.2 | 1.4 | 92 | 2000 | [^13^](#_ENREF_13) |
| ZnCo_2_O_4_/rGO | Symmetric | PVA/KOH | 0.4 | 143 | 11.44 | 1.382 | 93.4 | 5000 | [^14^](#_ENREF_14) |
| Pt/n-CNT@PANI | Symmetric | PVA–H_3_PO_4_ | 1 | 217.7 | 30.22 | 9.072 | 96 | 5000 | [^15^](#_ENREF_15) |
| Waste paper fibers-RGO–MnO_2_ | Symmetric | PVA–Na_2_SO_4_ | 0.8 | 220 | 19.6 | 2.4 | 85.3 | 2000 | [^16^](#_ENREF_16) |
| Porous carbon | Symmetric | PVA–KOH | 0.8 | 81.3 | 7.22 | 0.1 | ∼90.2 | 6000 | [^17^](#_ENREF_17) |
| NiCo_2_O_4_@PPy// activated carbon (AC) | Asymmetric | KOH/PVA | 1.6 | 165.4 | 58.8 | 10.2 | 89.2 | 5000 | [^18^](#_ENREF_18) |
| Graphene(IL-CMG)//RuO_2_–IL-CMG | Asymmetric | H_2_SO_4_/ PVA | 1.8 | 175 | 19.7 | 6.8 | - | - | [^19^](#_ENREF_19) |
| Ni–Co@Ni–Co LDH//carbon fibers | Asymmetric | PVA-KOH | 1.5 | 319 | 100 | 15 | 98.6 | 3000 | [^20^](#_ENREF_20) |
| CNT/polyaniline//CNT/MnO_2_/GR | Asymmetric | Na_2_SO_4_/ PVP | 1.6 | - | 24.8 |  |  |  | [^21^](#_ENREF_21) |
| Carbon aerogel//Co_3_O_4_ | Asymmetric | KOH-PVA | 1.5 | 57.4 | 17.9 | 7.5 | 85 | 1000 | [^22^](#_ENREF_22) |
| TiN@GNSs//Fe_2_N@GNSs | Asymmetric | LiCl/PVA | 1.6 | 60 | 15.4 | 6.4 | 98 | 20000 | [^23^](#_ENREF_23) |
| NiCo_2_O_4_/CC// porous graphene papers (PGP) | Asymmetric | LiOH/PVA | 1.8 | 71.32 | 60.9 | 11.36 | 96.8 | 5000 | [^24^](#_ENREF_24) |
| γ-MnS//eggplant derived AC (EDAC) | Asymmetric | KOH agar gel | 1.6 | 110.4 | 37.6 | 181.2 | 89.87 | 5000 | [^25^](#_ENREF_25) |
| CoS//AC | Asymmetric | PVA/KOH | 1.8 | 47 | 5.3 | 1.8 | 92 | 5000 | [^26^](#_ENREF_26) |
| CoMoO_4_@NiMoO_4_•xH_2_O// Fe_2_O_3_ | Asymmetric | PVA/KOH | 1.6 | 153.6 | 41.8 | 12 | 89.3 | 5000 | [^27^](#_ENREF_27) |
| CuS/3D graphene//3D graphene | Asymmetric | PVA/KOH | 1.6 | 32 | 5 | 3.2 | 77 | 1000 | [^28^](#_ENREF_28) |
| MnO_2_@PANI//3D graphene foam (GF) | Asymmetric | PVA/KOH | 1.5 | 95.3 | 37 | 4.0185 | 89 | 5000 | [^29^](#_ENREF_29) |
| NiCo_2_S_4_/polyaniline//AC | Asymmetric | PVA–KOH | 1.6 | 152.1 | 54.06 | 27.1 | 85.5 | 5000 | [^30^](#_ENREF_30) |
| NiCo-LDH//carbon nanorods | Asymmetric | KOH-PVA | 1.7 | 147.6 | 59.2 | 34 | 82 | 5000 | [^31^](#_ENREF_31) |
| Ni(OH)_2_/RGO/Ni//RGO aerogel/Ni | Asymmetric | PVA/KOH | 1.6 | 69 | 24.5 | 10.3 | 83 | 6000 | [^32^](#_ENREF_32) |
| rGO/CoAl-LDH//rGO | Asymmetric | PVA-KOH | 1.2 | 99.5 | 22.6 | 1.5 | 94 | 5000 | [^33^](#_ENREF_33) |
| NiS | Symmetric | PVA-LiClO_4_ | 1.2 | 55.83 | 9.3 | 0.67 | 90 | 1500 | [^34^](#_ENREF_34) |
| MnO_2_ | Symmetric | PVA-LiClO_4_ | 1.6 | 110 | 23 | 7.692 | 92 | 2200 | [^35^](#_ENREF_35) |
| V_2_O_5_ | Symmetric | PVA-LiClO_4_ | 1.8 | 358 | 43 | 3.604 | 88 | 1000 | [^36^](#_ENREF_36) |
| V_2_O_5_/ MWCNTs | Symmetric | PVA-LiClO_4_ | 1.8 | 629 | 72.07 | 8.4 | 96 | 4000 | [^37^](#_ENREF_37) |
| VS_2_/ MWCNTs | Symmetric | PVA-LiClO_4_ | 1.6 | 182 | 42 | 4.8 | 93.2 | 5000 | [^38^](#_ENREF_38) |
| CuS | Symmetric | PVA-LiClO_4_ | 1.6 | 172.5 | 12 | 1.75 | 93 | 2000 | [^39^](#_ENREF_39) |
| MWCNTs/ MnO_2_ | Symmetric | PVA-Na_2_SO_4_ | 1 | 204 | 28.33 | - | 80.36 | 2500 | [^40^](#_ENREF_40) |
| MnO_2_ | Symmetric | PVA-LiCl | 0.8 | 776 | - | - | 91 | 20000 | [^41^](#_ENREF_41) |
| Graphene/ polyaniline | Symmetric | PVA–H2SO4 | 0.8 | 665 | - | - | 100 | 10000 | [^42^](#_ENREF_42) |
| SWCNTs/ TiO_2_ | Symmetric | PVA–LiCl | 0.8 | 28 | - | 66.7 | 100 | 1000 | [^43^](#_ENREF_43) |
| MnO_2_//Fe_2_O_3_ | Asymmetric | Na_2_SO_4_/ CMC | 2 | 92 | 41.8 | 5.102 | 91 | 3000 | [^44^](#_ENREF_44) |
| β-MnO_2_//O-SnS | Asymmetric | PVA-LiClO_4_ | 1.6 | 122 | 29.8 | 1.25 | 95.3 | 5000 | [^45^](#_ENREF_45) |
| CeO_2_/MWCNTs | Symmetric | PVA-LiClO_4_ | 1.2 | 486.5 | 85.7 | 5.3 | 92.1 | 10000 | Present work |

**S5. Mass loading dependent electrochemical performance**

The optimum loading of CeO_2_ on MWCNTs was determined by optimizing deposition time for CBD method and results are shown in **Table S5** and **Figure S5**. In CBD, deposition time of 0.5 h results in less deposition of CeO_2_ nanoparticles over the MWCNTs surface, resulting low current response in CV. Again, over-growth and agglomeration occurred in case of engaging 1.5 h of deposition time, resisting the electrochemical reactions in spite of high mass loading which leads to low current response. Moreover, the film slowly peels off from the substrate surface due to over-deposition for involving deposition time more than 1.5 h. The deposition time of 1 h corresponds to optimum coating of CeO_2_ nanoparticles over the MWCNTs. The deposition of CeO_2_ nanoparticles over stable and conductive MWCNTs gives a unique morphology. Such surface modification facilitates high surface area that supplies maximum electroactive cavities for electrolyte ions to penetrate, resulting maximum electrochemical behavior.

Table S5: Mass loading as per the deposition time for CBD method.

| Deposition time (h) | Mass loading (mg/cm^2^) | Specific capacitance (F/g) |
| --- | --- | --- |
| 0.5 | 0.29 | 531.6 |
| 1.0 | 0.34 | 603.2 |
| 1.5 | 0.37 | 545.5 |


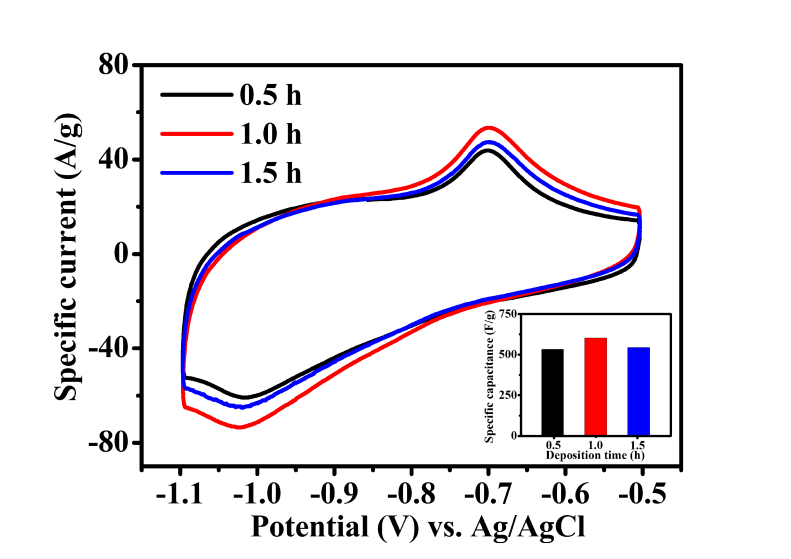


Figure S5: CV curves of CeO_2_/MWCNTs composite at scan rate of 100 mV/s for different deposition time, inset shows specific capacitance as a function of deposition time.

**References**

1. Vijayakumar, S., Lee, S.-H. & Ryu, K.-S. Hierarchical CuCo_2_O_4_ nanobelts as a supercapacitor electrode with high areal and specific capacitance. *Electrochim. Acta* **182**, 979-986 (2015).

2. Pandit, B. & Sankapal, B. R. Highly conductive energy efficient electroless anchored silver nanoparticles on MWCNTs as a supercapacitive electrode. *New J. Chem.* **41**, 10808-10814 (2017).

3. Dong, L. *et al.* Stacking up layers of polyaniline/carbon nanotube networks inside papers as highly flexible electrodes with large areal capacitance and superior rate capability. *J. Mater. Chem. A* **5**, 19934-19942 (2017).

4. Masikhwa, T. M., Madito, M. J., Bello, A., Dangbegnon, J. K. & Manyala, N. High performance asymmetric supercapacitor based on molybdenum disulphide/graphene foam and activated carbon from expanded graphite. *J. Colloid Interface Sci.* **488**, 155-165 (2017).

5. Zhao, D. *et al.* Highly Flexible and Conductive Cellulose-Mediated PEDOT:PSS/MWCNT Composite Films for Supercapacitor Electrodes. *ACS Appl. Mater. Interfaces* **9**, 13213-13222 (2017).

6. Chen, P., Chen, H., Qiu, J. & Zhou, C. Inkjet printing of single-walled carbon nanotube/RuO_2_ nanowire supercapacitors on cloth fabrics and flexible substrates. *Nano Res.* **3**, 594-603 (2010).

7. Hou, X. *et al.* Ultrathin ZnS nanosheet/carbon nanotube hybrid electrode for high-performance flexible all-solid-state supercapacitor. *Nano Res.* **10**, 2570-2583 (2017).

8. Javed, M. S. *et al.* High performance solid state flexible supercapacitor based on molybdenum sulfide hierarchical nanospheres. *J. Power Sources* **285**, 63-69 (2015).

9. Cao, J. *et al.* Three-dimensional graphene oxide/polypyrrole composite electrodes fabricated by one-step electrodeposition for high performance supercapacitors. *J. Mater. Chem. A* **3**, 14445-14457 (2015).

10. Liu, Y. *et al.* High-Performance Flexible All-Solid-State Supercapacitor from Large Free-Standing Graphene-PEDOT/PSS Films. *Sci. Rep.* **5**, 17045 (2015).

11. Li, S. *et al.* General Method for Large‐Area Films of Carbon Nanomaterials and Application of a Self‐Assembled Carbon Nanotube Film as a High‐Performance Electrode Material for an All‐Solid‐State Supercapacitor. *Adv. Funct. Mater.* **27**, 1700474 (2017).

12. Fan, Y.-M., Song, W.-L., Li, X. & Fan, L.-Z. Assembly of graphene aerogels into the 3D biomass-derived carbon frameworks on conductive substrates for flexible supercapacitors. *Carbon* **111**, 658-666 (2017).

13. Qiu, Y. *et al.* Flexible full-solid-state supercapacitors based on self-assembly of mesoporous MoSe_2_ nanomaterials. *Inorg. Chem. Front.* **4**, 675-682 (2017).

14. Kyu, M. I., Seonno, Y. & Jungwoo, O. Three‐Dimensional Hierarchically Mesoporous ZnCo_2_O_4_ Nanowires Grown on Graphene/Sponge Foam for High‐Performance, Flexible, All‐Solid‐State Supercapacitors. *Chem. Eur. J.* **23**, 597-604 (2017).

15. Wu, Y., Wang, Q., Li, T., Zhang, D. & Miao, M. Fiber-shaped Supercapacitor and Electrocatalyst Containing of Multiple Carbon Nanotube Yarns and One Platinum Wire. *Electrochim. Acta* **245**, 69-78 (2017).

16. Su, H. *et al.* Waste to wealth: A sustainable and flexible supercapacitor based on office waste paper electrodes. *J. Electroanal. Chem.* **786**, 28-34 (2017).

17. Li, X. *et al.* Hierarchical porous carbon from hazardous waste oily sludge for all-solid-state flexible supercapacitor. *Electrochim. Acta* **240**, 43-52 (2017).

18. Kong, D. *et al.* Three-Dimensional NiCo_2_O_4_@Polypyrrole Coaxial Nanowire Arrays on Carbon Textiles for High-Performance Flexible Asymmetric Solid-State Supercapacitor. *ACS Appl. Mater. Interfaces* **7**, 21334-21346 (2015).

19. Choi, B. G. *et al.* High performance of a solid-state flexible asymmetric supercapacitor based on graphene films. *Nanoscale* **4**, 4983-4988 (2012).

20. Yan, L. *et al.* Design of Hierarchical Ni-Co@Ni-Co Layered Double Hydroxide Core–Shell Structured Nanotube Array for High‐Performance Flexible All‐Solid‐State Battery‐Type Supercapacitors. *Adv. Funct. Mater.* **27**, 1605307 (2017).

21. Jin, Y., Chen, H., Chen, M., Liu, N. & Li, Q. Graphene-Patched CNT/MnO_2_ Nanocomposite Papers for the Electrode of High-Performance Flexible Asymmetric Supercapacitors. *ACS Appl. Mater. Interfaces* **5**, 3408-3416 (2013).

22. Liu, W., Li, X., Zhu, M. & He, X. High-performance all-solid state asymmetric supercapacitor based on Co_3_O_4_ nanowires and carbon aerogel. *J. Power Sources* **282**, 179-186 (2015).

23. Changrong, Z. *et al.* All Metal Nitrides Solid‐State Asymmetric Supercapacitors. *Adv. Mater.* **27**, 4566-4571 (2015).

24. Gao, Z., Yang, W., Wang, J., Song, N. & Li, X. Flexible all-solid-state hierarchical NiCo_2_O_4_/porous graphene paper asymmetric supercapacitors with an exceptional combination of electrochemical properties. *Nano Energy* **13**, 306-317 (2015).

25. Chen, T. *et al.* All-solid-state high performance asymmetric supercapacitors based on novel MnS nanocrystal and activated carbon materials. *Sci. Rep.* **6**, 23289 (2016).

26. Subramani, K., Sudhan, N., Divya, R. & Sathish, M. All-solid-state asymmetric supercapacitors based on cobalt hexacyanoferrate-derived CoS and activated carbon. *RSC Adv.* **7**, 6648-6659 (2017).

27. Wang, J. *et al.* Assembly of flexible CoMoO_4_@NiMoO_4_·xH_2_O and Fe_2_O_3_ electrodes for solid-state asymmetric supercapacitors. *Sci. Rep.* **7**, 41088 (2017).

28. Tian, Z., Dou, H., Zhang, B., Fan, W. & Wang, X. Three-dimensional graphene combined with hierarchical CuS for the design of flexible solid-state supercapacitors. *Electrochim. Acta* **237**, 109-118 (2017).

29. Ghosh, K., Yue, C. Y., Sk, M. M. & Jena, R. K. Development of 3D Urchin-Shaped Coaxial Manganese Dioxide@Polyaniline (MnO_2_@PANI) Composite and Self-Assembled 3D Pillared Graphene Foam for Asymmetric All-Solid-State Flexible Supercapacitor Application. *ACS Appl. Mater. Interfaces* **9**, 15350-15363 (2017).

30. He, X. *et al.* High-performance all-solid-state asymmetrical supercapacitors based on petal-like NiCo_2_S_4_/Polyaniline nanosheets. *Chem. Eng. J.* **325**, 134-143 (2017).

31. Wang, T. *et al.* 2-Methylimidazole-Derived Ni–Co Layered Double Hydroxide Nanosheets as High Rate Capability and High Energy Density Storage Material in Hybrid Supercapacitors. *ACS Appl. Mater. Interfaces* **9**, 15510-15524 (2017).

32. Lu, K. *et al.* Interfacial Deposition of Three-Dimensional Nickel Hydroxide Nanosheet-Graphene Aerogel on Ni Wire for Flexible Fiber Asymmetric Supercapacitors. *ACS Sustain. Chem. Eng.* **5**, 821-827 (2017).

33. Zhang, R. *et al.* Mesoporous graphene-layered double hydroxides free-standing films for enhanced flexible supercapacitors. *Chem. Eng. J.* **289**, 85-92 (2016).

34. Patil, A. M., Lokhande, A. C., Chodankar, N. R., Kumbhar, V. S. & Lokhande, C. D. Engineered morphologies of β-NiS thin films via anionic exchange process and their supercapacitive performance. *Mater. Des.* **97**, 407-416 (2016).

35. Chodankar, N. R., Dubal, D. P., Gund, G. S. & Lokhande, C. D. A symmetric MnO_2_/MnO_2_ flexible solid state supercapacitor operating at 1.6V with aqueous gel electrolyte. *J. Energy Chem.* **25**, 463-471 (2016).

36. Pandit, B., Dubal, D. P. & Sankapal, B. R. Large scale flexible solid state symmetric supercapacitor through inexpensive solution processed V_2_O_5_ complex surface architecture. *Electrochim. Acta* **242**, 382-389 (2017).

37. Pandit, B., Dubal, D. P., Gómez-Romero, P., Kale, B. B. & Sankapal, B. R. V_2_O_5_ encapsulated MWCNTs in 2D surface architecture: Complete solid-state bendable highly stabilized energy efficient supercapacitor device. *Sci. Rep.* **7**, 43430 (2017).

38. Pandit, B., Karade, S. S. & Sankapal, B. R. Hexagonal VS_2_ Anchored MWCNTs: First Approach to Design Flexible Solid-State Symmetric Supercapacitor Device. *ACS Appl. Mater. Interfaces* **9**, 44880-44891 (2017).

39. Patil, A. M. *et al.* Interior design engineering of CuS architecture alteration with rise in reaction bath temperature for high performance symmetric flexible solid state supercapacitor. *J Ind. Eng. Chem.* **46**, 91-102 (2017).

40. Chodankar, N. R., Ji, S.-H. & Kim, D.-H. Low-cost superior symmetric solid-state supercapacitors based on MWCNTs/MnO_2_ nanocomposite thin film. *J. Taiwan. Inst. Chem. Eng.* **80**, 503-510 (2017).

41. Soni, R., Raveendran, A. & Kurungot, S. Grafoil-Scotch tape-derived highly conducting flexible substrate and its application as a supercapacitor electrode. *Nanoscale* **9**, 3593-3600 (2017).

42. Li, K., Liu, J., Huang, Y., Bu, F. & Xu, Y. Integration of ultrathin graphene/polyaniline composite nanosheets with a robust 3D graphene framework for highly flexible all-solid-state supercapacitors with superior energy density and exceptional cycling stability. *J. Mater. Chem. A* **5**, 5466-5474 (2017).

43. Chen, C. *et al.* Foldable All‐Solid‐State Supercapacitors Integrated with Photodetectors. *Adv. Funct. Mater.* **27**, 1604639 (2017).

44. Gund, G. S. *et al.* Low-cost flexible supercapacitors with high-energy density based on nanostructured MnO_2_ and Fe_2_O_3_ thin films directly fabricated onto stainless steel. *Sci. Rep.* **5**, 12454 (2015).

45. Patil, A. M., Lokhande, V. C., Patil, U. M., Shinde, P. A. & Lokhande, C. D. High Performance All-Solid-State Asymmetric Supercapacitor Device Based on 3D Nanospheres of β-MnO_2_ and Nanoflowers of O-SnS. *ACS Sustain. Chem. Eng.* **6**, 787-802 (2018).
